# How could local environmental innovations become a national policy?—A qualitative comparative study on the factors influencing the diffusion of bottom-up environmental policy innovations

**DOI:** 10.1371/journal.pone.0325811

**Published:** 2025-06-25

**Authors:** Jiliang Zhang, Shuya Xing

**Affiliations:** 1 School of Political Science and Public Administration, Shandong University, Shandong, China; 2 School of Humanities and Law, Northeastern University, Shenyang, China; Zhejiang Gongshang University, CHINA

## Abstract

Innovation diffusion in China’s local environmental governance occurs both horizontally and vertically from the bottom up. This process is critical to improving environmental policies and advancing our understanding of how local innovations spread. To explore this phenomenon, the study applies fuzzy-set qualitative comparative analysis (fsQCA) to 17 environmental cases. It examines five key factors: central agenda setting, recognition by central government departments, focal events, policy innovation costs and difficulties, and diffusion among local governments. The findings indicate that national policy integration is driven by five distinct pathways, rather than by any single factor. These pathways fall into three broader models: Central Guidance, Drive by Centre and Society, and Local Autonomy. This study introduces a new analytical framework to explain the vertical diffusion of local environmental innovations within China’s governance structure. It deepens our understanding of innovation diffusion in local governance and provides practical guidance for both central and local governments. Additionally, it lays a foundation for future research on environmental policy diffusion.

## Introduction

As global challenges such as climate change, water pollution, air pollution, and desertification become increasingly severe, there is growing concern about environmental issues. In particular, much attention is focused on how to effectively address these challenges. A key area of research that is closely related to these environmental problems centers on various forms of governance, including adaptive governance [1–3], participatory and collaborative governance [4,5], deliberative governance [6,7], good governance [8,9], institutional governance [10,11], meta-governance [12,13], and transnational network governance [14,15]. Although the various forms of environmental governance reflect the public’s attention to environmental protection, the perspective of policy innovation diffusion can still offer new approaches to understanding and addressing environmental issues. Scholars have increasingly recognized the theoretical and practical significance of governance innovation and its diffusion, with much of the research focusing on horizontal diffusion mechanisms [16,17], top-down vertical guidance mechanisms [18,19], local government behavior [20–22], and the individual innovation motivations of officials [23–25]. Although some studies address the vertical diffusion dimension of environmental governance innovation, they either focus on top-down coercive diffusion mechanisms or, while exploring bottom-up diffusion, primarily examine cases of environmental innovation diffusion in federal countries. Overall, there remains a lack of in-depth research on bottom-up vertical diffusion mechanisms in unitary states. Unlike federal or confederal systems, unitary systems are characterized by the centralization of political authority. In unitary systems, the central government holds supreme power, and sub-national units—if they exist—operate only through powers granted or delegated by the center. This stands in contrast to federal systems, where authority is constitutionally divided between the central and sub-national levels, and to confederacies, where the central institutions are subordinate to the sovereign member states [26].

This study uses China as a case to explore how local governments innovate in environmental governance based on local needs [27], how these innovations spread across different levels of government, and how they are eventually adopted by the central government as formal national policies. The findings show that bottom-up diffusion of environmental innovations occurs not only in federal systems but also in unitary states. This research enriches our understanding of bottom-up diffusion models and sheds light on the flexibility of China’s political system. It also provides practical insights into how formal systems or institutions can be established at the national level to promote successful innovative practices. This study begins by reviewing existing research on the limited vertical diffusion of local environmental innovations in China. Building on this, an analytical framework is developed by incorporating relevant studies from other countries. Next, the fsQCA method is applied to empirically analyze 17 cases of vertical diffusion of environmental innovations. Finally, we summarize the paper and present future research prospects.

## Literature review

Since the late 1960s, policy innovation diffusion has been a key topic in political science and public policy research, with Walker pioneering its study [28]. Scholars have since identified four primary mechanisms of diffusion: competition, imitation, learning, and coercion [29]. In the 21st century, research has expanded to examine the content (what), location (where), initiators (who), methods (how), and timing (when) of diffusion, significantly enriching our understanding of this field [30]. Despite significant advancements, research on bottom-up vertical policy innovation diffusion remains underexplored, particularly in contexts like environmental governance in unitary states such as China.

Existing international research on vertical policy innovation diffusion focuses on three main aspects. First, the completeness of vertical diffusion examines how much of the original policy innovations are retained or transformed when adopted by higher-level governments. Some policies are fully adopted, preserving all original provisions [31], while others are partially adopted or limited to the diffusion of ideas without implementing the original policies [32–34].

Second, various factors influence the vertical diffusion of policy innovations, including policy attributes and the role of agents. Non-distributive policies are more likely to diffuse vertically due to their lower potential for political disputes [35]. Agents such as NGOs and local governments play crucial roles; NGOs often lobby central governments to adopt local innovations, while horizontal diffusion among local governments can signal the advantages of these policies to higher authorities [36,37]. Additionally, international competition may pressure central governments to adopt local innovations for a competitive edge [38].

Third, the mechanisms of vertical diffusion highlight the dynamic processes through which policies spread across levels of government. Imitation mechanisms often drive diffusion when successful policies are emulated by governments with similar contexts [39]. Competition mechanisms operate among peer governments, including federal governments competing internationally or neighboring states with comparable economies [38,40,41]. Pressure mechanisms, such as federal intervention or external influences from the international community, also play a significant role in vertical diffusion [38,42].

These studies primarily build on the policy experiences of Western countries, particularly the United States and Brazil. They focus on vertical policy diffusion in federal systems, particularly examining the extent of diffusion, the influencing factors, and the mechanisms behind it. These aspects illustrate the importance of vertical diffusion in the process of policy innovation. However, they largely focus on public health and rarely address environmental governance. As a unitary state, China’s governance structure is highly centralized, relying predominantly on top-down directives. This poses unique challenges for understanding bottom-up policy diffusion, in the environmental sector, where local innovations must navigate a hierarchical political system. Analyzing environmental policy diffusion in China thus requires adapting insights from federal contexts to better account for its centralized power dynamics and local-central interactions.

China’s local environmental governance operates under a system that adheres to centralized leadership while actively leveraging initiative and proactivity of local governments. The various innovations in environmental governance undertaken by local governments reflect this proactive engagement. In practice, there are instances where local environmental governance innovations are integrated into the national environmental policy system by the central government [43]. However, a review of research in China reveals that mainstream academic attention focuses primarily on the horizontal diffusion of local environmental governance innovations [44–46], with relatively little research on vertical diffusion, particularly at the bottom-up level [47,48].

Research on the vertical diffusion of local environmental governance innovations explores the factors influencing such diffusion from five perspectives: policy attributes, institutionalization, state-society relations, policy experimentation, and comprehensive analysis. Firstly, from the perspective of policy attributes, researchers analyze the characteristics of local government environmental innovation policies and investigate how certain policy innovations achieve vertical diffusion by aligning with national governance needs when specific “windows of opportunity” open up in terms of value acceptability and technical feasibility [49]. Secondly, existing studies have taken a comprehensive institutional perspective to develop a new framework for analyzing vertical diffusion. They use theory-driven process tracing to examine specific cases, aiming to understand how diffusion occurs between central and local governments in China. Studies on the River Chief System, for example, have explored the mechanisms through which local environmental policy innovations diffuse vertically and evolve across different levels of government [50–53]. Thirdly, from the perspective of state-society relations [54–56], researchers highlight the role of non-governmental organizations (NGOs) in driving the horizontal and vertical diffusion of local environmental governance innovations, systematically analyzing the current status, constraints, and countermeasures of bottom-up environmental NGOs. Fourthly, from a policy experimentation perspective [57], the dynamic process of vertical diffusion from “point to surface” of local environmental innovation policies in China is explored, illustrating the process from central to local level, and then vice versa [58]. Lastly, some studies take a comprehensive actor–institution–mechanism perspective. They examine how multiple actors such as the CPPCC and the People’s Congress, national environmental governance institutions, and the competitive–cooperative relationships among local governments help promote the vertical diffusion of local environmental governance innovations [43].

Existing studies provide insights for understanding the vertical diffusion of local environmental governance innovations within China’s decentralized administrative structure. However, they lack a perspective that focuses specifically on the diffusion of policy innovations, particularly vertical diffusion. The (vertical) policy innovation diffusion perspective allows for a more comprehensive understanding of the factors influencing the vertical diffusion of local environmental governance innovations from multiple dimensions. Moreover, from a methodological perspective, most existing studies on the vertical diffusion of local environmental policy innovations tend to use single-case analysis. While single-case studies facilitate in-depth analysis of environmental policy diffusion [49], few studies adopt a comparative approach to analyze the factors influencing the vertical diffusion of different local environmental innovation projects.Therefore, this paper employs the theoretical perspective of vertical policy innovation diffusion and utilizes the fsQCA research method. Through multi-case comparative analysis, it systematically examines the factors influencing the upward diffusion of environmental practices, elucidating how local environmental governance innovations can become national policies.

### Analytical framework

This study draws on Rogers’ basic framework for the horizontal diffusion of innovation, which comprises four key elements: innovation, communication channels, time, and social systems [59]. The innovation elements include relative advantage, complexity, compatibility, observability, and visibility. In terms of communication channels, mass media is considered the most effective method for disseminating innovation information. This model has had a wide impact, as many scholars have continually refined, supplemented, and validated it through both theoretical and empirical research [60–63]. However, this framework has its limitations, as it primarily focuses on horizontal innovation diffusion and lacks considerations for vertical innovation diffusion. Besides, the focus of this research is more on countries and regions outside of China. Therefore, this study draws on international research on vertical diffusion of innovation and incorporates the Chinese policy context. It builds a comprehensive analytical framework based on two dimensions: “central and societal conditions” and “local conditions.” The former corresponds to Rogers’ emphasis on the social system and communication channels. It highlights the role of the broader institutional environment and information flows in shaping diffusion pathways. The latter aligns with innovation characteristics and local social structures, emphasizing local governments’ adaptive adoption and internal motivation for policy innovation. This framework is designed to analyze the factors influencing the bottom-up vertical diffusion of local environmental policy innovations in China. It should be noted that, given the constraints imposed by the political structure, obtaining first-hand interview data from officials at or above the provincial level is particularly challenging. Consequently, agency-related factors are limited in the selection of variables in this study.

The central and societal conditions for the vertical diffusion of environmental policy innovation include central agenda-setting [59,64,65], the recognition by relevant central government departments [66,67], and focal events [65,68,69]. Central agenda-setting refers to the intentional efforts by central leaders to address specific issues, as mentioned in their speeches and five-year plans, laying the groundwork for recognition by relevant central government departments. Recognition by central government departments involves the issuance of official documents or the initiation of relevant pilot projects by national ministries and commissions (such as the Ministry of Ecology and Environment, the Ministry of Natural Resources, and the Ministry of Agriculture and Rural Affairs) through annual ecological and environmental protection plans, thereby granting a degree of legitimacy to the policy innovation in terms of policy concepts [49]. Focal events refer to social incidents capturing the attention of the public and media, which are more likely to occur after gaining recognition from central government departments. Consequently, the allocation of attention by the central government to environmental governance is often a theoretical prerequisite for environmental issues to enter the agenda, leading to policy formulation and implementation [70].

However, the limited attention of the central government can be easily squeezed by problems arising from preferential mechanisms [71]. Once attention shifts away from problem mechanisms, it will become a challenge to find solutions [72]. At this juncture, focal events in society can influence the recognition by central government departments and even the allocation of attention by the central government. If the government’s allocation of attention signifies its proactive concern for a particular environmental agenda [73], then focal events demonstrate the need for the government to actively respond to the attention of the public and media, transforming the public agenda into a government agenda and proactively formulating and implementing corresponding policies [74]. The allocation of attention by the public and media provides a bottom-up political opportunity structure for the vertical diffusion of local environmental policy innovation.

The local conditions for the vertical diffusion of environmental policy innovation encompass two main aspects: the cost and complexity of policy innovation [66,75,76] and the diffusion of policy innovation among local governments [54,56].Firstly, the cost and complexity of environmental policy innovation significantly influence its diffusion scope. Generally speaking, the more costly an environmental policy innovation is, and the more it requires the cooperation of multiple departments, the more limited the diffusion of the innovation will be. Conversely, lower costs and simpler implementation requirements facilitate the wider diffusion [77]. Secondly, the diffusion of policy innovation among local governments refers to the phenomenon where, after an environmental innovation policy achieves success and exerts impact in one locality, other local governments begin to imitate, learn from, and adopt similar policy measures. This indicates the broad adaptability of the environmental innovation.

**Fig 1:**
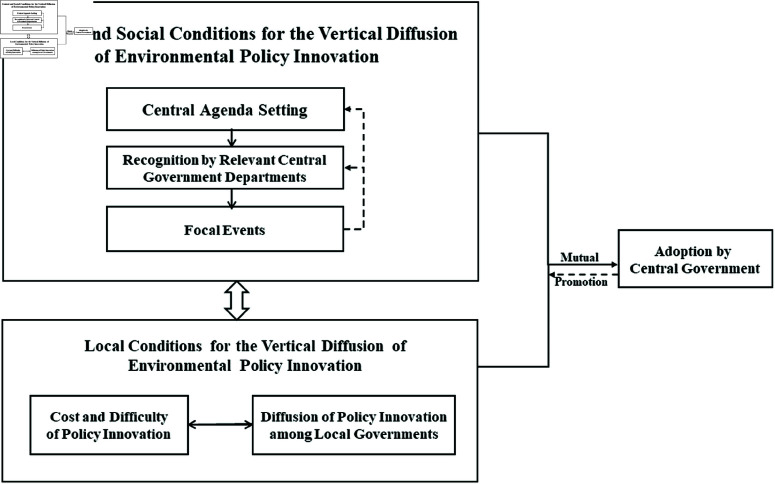
Analytical framework.

There is also a close logical relationship between these two local factors. Specifically, the cost and complexity of policy innovation are theoretical guarantees for its initial success, which in turn promotes broader diffusion among local governments. The success of policy innovation in the original location provides a model for other local governments to learn from and imitate, making the assessment of the innovation’s cost and complexity a critical step in its diffusion among local governments. These conditions lay the foundation for elevating local environmental practices to the national policy, as illustrated in Fig 1.

In summary, the more attention the central government, relevant central departments, and the mass media pay to an environmental issue, the higher its priority becomes [78]. This heightened priority, in turn, exerts pressure on both central and local departments to address the issue promptly. Consequently, this attention creates a “policy window of opportunity” from both top-down and bottom-up directions, enabling certain local environmental innovation policies to become national policies, thereby achieving top-down vertical diffusion [36].This process aligns closely with Rogers’ innovation diffusion framework. In particular, the cost and complexity of policy innovation correspond to the “innovation” element; focal events reflect the “communication channels” element; the central agenda setting, the recognition by relevant central departments, and the diffusion of policy among local governments pertain to the “social system” element; and the time required for bottom-up policy diffusion corresponds to the “time” element.

## Methods

### Research methods

The Qualitative Comparative Analysis (QCA) method, introduced by sociologist Charles Ragin in 1987, employs a holistic and set-theoretic framework, emphasizing the complexity of causal relationships between conditions and outcomes and is particularly useful for exploring cases where multiple factors converge to produce a single outcome. For the latest advancements in this methodology, refer to the research conducted by Patrick A. Mello and colleagues [79]. Traditional quantitative analysis presumes mutual independence among independent variables, a unidirectional linear relationship, and causal symmetry. While this approach is adept at identifying the marginal “net effect” of dependent variables, it faces limitations in accounting for interdependencies among independent variables and in elucidating complex causal relationships [80]. Furthermore, while traditional qualitative analysis provides detailed insights into individual cases, it is often constrained when applied across multiple cases. In contrast, the objective of QCA is to transcend the dichotomy between qualitative and quantitative research paradigms. QCA integrates these two approaches by adopting a case-centered methodology that ensures a close linkage between the research process and the cases analyzed [81]. Utilizing set theory and Boolean algebra, QCA explores configurational effects among multiple variables, allowing for “equifinality”—a concept in which various paths lead to the same outcome [82].At present, QCA has developed four relatively mature analytical approaches: crisp-set QCA (csQCA), fuzzy-set QCA (fsQCA), multi-value QCA (mvQCA), and temporal QCA (TQCA) [79]. Among them, fsQCA allows for the calibration of each variable and handles continuous variables by generating fuzzy set membership scores. It builds a truth table and identifies multiple combinations of conditions that lead to the same outcome.

This study employs fsQCA (fuzzy-set Qualitative Comparative Analysis) for three main reasons: First, Complexity of Influencing Factors: The factors influencing the diffusion of local environmental innovations go beyond simple linear correlations. Qualitative Comparative Analysis can reveal the configurational effects of multiple conditional variables. Second, Sample Size: The number of cases of local autonomous environmental innovation practices is relatively small, which makes it difficult to meet the huge requirements of the sample size in the quantitative analysis. fsQCA is particularly suitable for comparative analysis of small to medium-sized samples [83].Third, Variable Nature: There are continuous variables among the selected variables. fsQCA can handle variations in degree and issues of partial affiliation [79], allowing for the identification of subtle effects of variables across different degrees of change.

### Case selection

This study follows the principles of similarity and maximum differentiation in fsQCA case selection [84] and meets the basic requirements of case study methodology. Based on these principles, it collected 24 cases of local environmental innovation practices carried out by local governments before the issuance of relevant central policies. These cases were identified through multiple sources, including government websites, China Environmental Statistics Yearbook, Peking University’s legal database, and Baidu Baike, and were used to establish a pool of potential cases. In selecting cases, this study strictly adheres to the guidelines set by Robert Yin [85]. These guidelines include using multiple data sources, establishing a case study database, creating a chain of evidence, and carefully utilizing electronic resources. Since the innovations examined here are bottom-up, we rigorously excluded cases initiated or piloted by the central government [86], retaining only those spontaneously adopted by local governments and later accepted by the central government. This process resulted in the selection of 17 cases of independent environmental practices conducted by the local government for in-depth analysis. Finally, we examined them from three perspectives: natural resources, social activities, and mixed categories. Supporting materials were retrieved by using web crawlers from the China National Knowledge Infrastructure (CNKI) newspaper database and various policy documents. Through triangulation and verification [87], we established a highly credible database of local environmental policy innovation cases. The results are summarized in Table 1.

**Table 1:** The execution time.

No.	Year	Name of environmental practice	Initial location	Category
1	1956	Policy on the Construction and Management of Nature Reserves	Dinghu District, Zhaoqing City, Guangdong Province	Natural Resources
2	1987	Water Resource Management Policy	Beijing-Tianjin Region	
3	2000	Water Rights Trading System	Dongyang and Yiwu, Zhejiang Province	
4	2003	River Chief System	Changxing County, Zhejiang Province; Eryuan County, Yunnan Province	
5	2009	Lake Chief System	Huanggang City, Hubei Province	
6	2016	Forest Chief System	Fuzhou City, Jiangxi Province	
7	2020	Integrated Protection of Mountains, Rivers, Forests, Fields, Lakes, Grass, and Sand	Guilin City, Guangxi Province	
8	1987	Paid Use and Trading System for Emission Rights	Minhang District, Shanghai	Social Activities
9	1990s	Toilet Revolution	Tourist cities such as Beijing	
10	1991	Construction of Environmental Monitoring and Data Sharing Platforms	Shenyang, Taiyuan, Nanchang	
11	1996	Waste Sorting	Beijing	
12	2002	Vertical Management of Environmental Protection	Yan’an City, Shaanxi Province	
13	2003	“Thousand Villages Demonstration, Ten Thousand Villages Renovation” Project	Zhejiang Province	
14	2006	Automatic Pollution Source Monitoring System	Shanxi Province	
15	2010	Ecological Compensation System	Suzhou City, Jiangsu Province	
16	2008	Beautiful Countryside Construction	Anji County, Zhejiang Province	Mixed
17	2012	Rural Complex	Jiangsu Province	

### Variable setting and construction of the truth table

Based on the analytical framework of “central and societal conditions-local conditions,” this study uses the literature synthesis method to determine the conditional variables. In qualitative comparative analysis (QCA), there are five main methods for setting variables: problem-oriented, existing literature synthesis, theoretical perspective, research framework, and empirical summary. This study employs the literature synthesis method for variable setting. Specifically, the central and societal conditions dimension for the vertical diffusion of environmental innovation policies is transformed into three variables: central agenda setting, recognition by relevant central government departments, and focal events. The local conditions dimension for the vertical diffusion of environmental policy innovation is transformed into two variables: the cost and complexity of local environmental policy innovation and the diffusion of policy innovation among local governments. The outcome variable is transformed as the time required for bottom-up policy diffusion of local environmental policy innovation.

#### Outcome variable and calibration.

This study uses the time required for local environmental innovation to be promoted nationwide or piloted by central ministries as a measure of the continuous variable, because the duration required for a policy to progress from local innovation to central adoption serves as an indicator of the speed of policy diffusion. In the fsQCA, the calibrate(x, n1, n2, n3) function is employed to directly calibrate the initial values of the continuous variable. Here, x represents the variable. The n1, n2, and n3 are set as thresholds corresponding to fuzzy affiliation scores of 0.95, 0.5, and 0.05, respectively, transforming them into fuzzy affiliation values ranging from 0 to 1. During calibration, to avoid a fuzzy membership score of 0.5 for case conditions, the approach suggested by Fiss is adopted [88], where an increment of 0.001 is added to the base value of 0.5. Since the closer the outcome variable is to 1, the closer it is to a successful result, whereas in this paper the longer the diffusion takes, the closer the calibrated value will be to 1. Therefore, the complement of the directly calibrated value is used as the final data.

#### Conditional variables and assignment.

The conditional variables in this study are the factors influencing the time required for bottom-up innovation diffusion, as shown in Table 2.

**Table 2:** Variable selection and assignment.

Variable category		Variable selection	Data source	Variable measurement
Conditional Variables	Central and Societal Conditions	Central Agenda Setting (CAS)	Government websites, national and provincial policy documents	1: Involves both the five-year plan and attention from leaders; 0.501: Involves either the five-year plan or leader attention; 0: Involves neither the five-year plan nor leader attention.
		Recognition by Relevant Central Government Departments (RRDCG)	National ministry websites, Peking University legal database	1: National ministries endorse and pilot the local practice; 0: No endorsement or piloting by national ministries.
		Focal Events (FE)	CNKI newspaper database, Baidu Baike database	1: Social opinion event occurred; 0: No social opinion event occurred.
	Local Conditions	Cost and Difficulty of Policy Innovation (CDPI)	“China Environmental Statistics Yearbook,” Peking University legal database	1: Meets all three conditions of low cost, few personnel needed, and no need for cooperation with other institutions; 0.67: Meets two of the three conditions; 0.33: Meets one of the three conditions; 0: High cost, many personnel needed, and requires cooperation with other institutions.
		Diffusion of Policy Innovation among Local Governments (DPILG)	Baidu Baike database, national and provincial policy documents	Number of provincial, sub-provincial, and prefecture-level administrative units adopting the innovation before it becomes national policy. If the highest adopting unit is provincial, anchor points are set at 1 and 0.67. If sub-provincial, anchor points are 0.66 and 0.33. If city-level, anchor points are 0.33 and 0.
Outcome Variable		Time Required for Bottom-Up Policy Diffusion (TRBPD)	Government websites, national and provincial policy documents	The difference between the year the policy is fully promoted and the year it originated (for policies not fully promoted, the year of ministry piloting is used)

First, Central Agenda Setting. Central Agenda Setting refers to the central government’s intention to address specific issues, aiming to promote the implementation of central policies and achieve institutional goals at a macro level. The five-year plans reflect the central government’s strategic priorities. When a local practice by the nation is highlighted in the National People’s Congress, Party Congress, or State Council directives, it is considered that the environmental policy has gained attention from central leaders. The allocation of attention by the central government can significantly advance the elevation of local environmental innovation policies into central policies. When the central government pays special attention to a local environmental innovation policy, it not only elevates the policy’s status but also triggers broader policy discussions. This influence can rapidly push the local policy onto a higher-level policy-making agenda, facilitating its widespread implementation nationwide. In this study, cases that meet both the five-year plan and leader attention criteria are assigned a value of 1; if one criterion is met, a value of 0.501 is assigned; if neither criterion is met, a value of 0 is assigned.

Second, Recognition from Relevant Departments of the Central Government.Recognition from relevant central government departments not only motivates the government to take proactive actions but also sends a strong signal nationwide, promoting the rapid formulation and implementation of environmental policies. The annual ecological environment protection plan reflects the attitude of national ministries towards the environment at the tactical level, and pilot programs mandated by offices and national ministries further validate the acceptance of local environmental practices by central government departments. In this study, cases mentioned in the annual ecological environment protection plan and accompanied by policy documents requiring pilot programs are assigned a value of 1; if no pilot programs are conducted, the value of 0 is assigned.

Third, Focal Event. Focal events can serve as catalysts for policy diffusion because they attract widespread attention and discussion, resonating among the public, media, and policymakers. This resonance can swiftly shift relevant environmental issues from the public agenda to the government agenda and transform general government agendas into specific environmental decisions. This study takes the social opinion event as an indicator to measure focal events. If a social event occurred within two years before the local practice became national policy, a value of 1 is assigned; if no such event occurred, a value of 0 is assigned.

Fourth, The Cost and Difficulty of Policy Innovation. The attributes of policy innovation can gauge the horizontal and vertical diffusion range of an environmental innovation policy. Given that implementing such a policy requires certain human, material, and financial resources as well as support from relevant departments, the more support required, the more costly and difficult the environmental policy innovation will be, making it more challenging to diffuse horizontally and vertically. Referring to previous research [89], this study uses three indicators to measure the attributes of policy innovation: the cost, the number of personnel required, and the need for cooperation with other institutions. If an environmental policy meets all three conditions (low cost, few personnel required, no need for cooperation), it is assigned a value of 1; if it meets two of the three conditions, a value of 0.67 is assigned; if it meets only one condition, a value of 0.33 is assigned; if none of the conditions are met, a value of 0 is assigned.

Fifth,The Diffusion of Policy Innovation among Local Governments. Before a ministry or the central government endorses and promotes an environmental innovation policy, if other local governments have already spontaneously learned and adopted the policy, it indicates that the innovation has achieved success not only in the original local government but also in other provinces. By examining the number of provincial, sub-provincial, and prefecture-level governments adopting each environmental policy during the period from the initiation of its innovation to its adoption into a national policy, the original data on horizontal diffusion is obtained. To reflect the different levels of diffusion, the highest diffusion level is used. Therefore, cases where the highest diffusion level reaches the provincial level are assigned values between 0.67 and 1, cases reaching the sub-provincial level are assigned values between 0.33 and 0.66, and cases reaching the prefecture level are assigned values between 0 and 0.32. Since the data are continuous variables, data normalization is used to transform the original data into corresponding ranges for provincial, sub-provincial, and prefecture-level administrative units, completing the calibration of the original data.

After assigning values to the 17 cases based on the above coding principles, the new calibrated variables are obtained, and the truth table is constructed, as shown in Table 3.

**Table 3:** Truth table.

Case Number	TRBPD	CAS	RRDCG	FE	CDPI	DPILG
1	0	1	1	1	1	0.06
2	0.46	0.501	1	0	1	0.76
3	0.501	1	0	1	0.33	0.03
4	0.57	0.501	1	0	0.67	0.72
5	0.85	0.501	0	0	0.67	0.67
6	0.94	1	1	1	0.33	0.32
7	0.98	1	1	1	0.67	1
8	0.3	1	0	1	0.33	0.67
9	0.12	0.501	0	0	0.33	0.66
10	0.19	1	1	1	0.67	0.81
11	0.19	1	1	1	0.67	0
12	0.501	0.501	0	0	0.33	0.33
13	0.43	0.501	0	0	0.33	0.72
14	0.7	1	0	1	1	0
15	0.7	0	0	0	1	0.81
16	0.501	1	1	1	0.67	0
17	0.93	0.501	0	0	1	0.72

## Results

### Necessity analysis of single variables

The necessity analysis of single variables is the first step in fsQCA analysis. A necessary condition is a single factor that is always present when the outcome occurs [90]. This is typically assessed through consistency, which reflects whether cases with the same conditions share a common conclusion [91]. When the consistency of the antecedent condition is greater than 0.9, the condition passes the consistency test and can be considered a necessary condition for the outcome. When the consistency of the antecedent condition is greater than 0.9, the condition passes the consistency test and can be considered a necessary condition for the outcome. The simplified formula for consistency is shown as (1).

$$Consistency(X_{i}\leq Y_{i})=\frac{\sum \min X_{i},Y_{i}}{\sum X_{i}},$$ (1)

Additionally, coverage can be used to evaluate the explanatory power of the antecedent conditions or configurations for the outcome variable. The higher the coverage is, the stronger the explanatory power of the variable will be. The simplified formula for coverage is shown as (2).

$$Coverage(X_{i}\leq Y_{i})=\frac{\sum \min X_{i},Y_{i}}{\sum Y_{i}},$$ (2)

The consistency results indicate that the consistency of all five selected conditional variables is less than 0.9. This suggests that central agenda setting, recognition by relevant central government departments, focal events, the cost and difficulty of policy innovation, and the diffusion of policy innovation among local governments are not necessary conditions for reducing the time required for bottom-up diffusion. Details are shown in Table 4.

**Table 4:** Univariate necessity analysis.

Conditional variable	Time required for bottom-Up policy diffusion	
	Consistency	Coverage
Central Agenda Setting	0.825454	0.584952
Recognition by Relevant Central Government Departments	0.432246	0.478875
Focal Events	0.485389	0.478000
Cost and Difficulty of Policy Innovation	0.826018	0.665545
Diffusion of Policy Innovation Among Local Governments	0.656663	0.702899

### Sufficiency analysis of conditional configurations

Based on the discussion of the necessity conditions of a single variable, this study further explores the adequacy of conditional configurations, using the “consistency” indicator for evaluation. The truth table, derived from the fuzzy affiliation score matrix, may contain contradictory configurations and logical remainders, which could affect the reliability of the results and the validity of causal inferences [92,93]. Therefore, it is essential to optimize the truth table. Following standard research conventions, the consistency threshold for determining the adequacy of conditional variables should not be lower than 0.75 [84]. This study sets the consistency threshold for sufficiency analysis at 0.8 and the case frequency threshold at 1, excluding logical remainders to obtain three forms of solutions: complex, simplified, and intermediate solutions. The parsimonious solution disregards the empirical plausibility of logical remainders. In contrast, the complex solution emphasizes the role of causal conditions. Only the intermediate solution preserves all necessary conditions identified in the analysis. Referring to existing studies [88,89], this research prioritizes the intermediate solution in the fsQCA adequacy analysis results. By comparing the intermediate and simplified solutions, the core and peripheral conditions leading to the outcome are further distinguished [79]. Therefore, this study takes the intermediate solution as the core basis, supplemented by the simplified solution in the specific result analysis, as shown in Table 5 and Table 6.

**Table 5:** Intermediate solution.

	Raw coverage	Unique coverage	Consistency
Cas ^*^ rrdcg^*^ CDPI^*^ DPILG	0.229268	0.019294	0.829049
Cas^*^fe^*^ CDPI^*^ DPILG	0.246079	0.155703	0.865476
Cas^*^ rrdcg^*^fe^*^ DPILG	0.169356	0.078980	0.652609
RRDCG^*^ fe^*^cdpi^*^DPILG	0.323818	0.122757	0.899687
Cas^*^ FE^*^CDPI^*^DPILG	0.309489	0.108428	0.916166
solution coverage:	0.776599		
solution consistency:	0.840107		

**Table 6:** Parsimonious solution.

	Raw coverage	Unique coverage	Consistency
CDPI^*^ DPILG	0.416563	0.155704	0.835294
FE^*^CDPI	0.463726	0.084622	0.077107
rrdcg^*^ DPILG	0.361277	0	0.729385
RRDCG^*^cdpi	0.497574	0	0.828947
solution coverage:	0.808078		
solution consistency:	0.753 895		

Based on the output results, five distinct conditional combination approaches can be observed. The overall coverage and consistency of this model are 0.776599 and 0.840107, respectively, indicating that approximately 78% of the cases can be explained by all the conditional combinations, demonstrating a strong explanatory power. According to Boolean algebra rules, approaches two and three can be simplified to: Central Agenda Setting ^*^ Focal Events ^*^ Diffusion of Policy Innovation Among Local Governments, while approaches four and five can be simplified to: Focal Events ^*^ Cost and Difficulty of Policy Innovation ^*^ Diffusion of Policy Innovation Among Local Governments. Based on the existence and combination of various factors (central, societal, or local) that serve as prerequisites for policy diffusion, this study categorizes the pathways of vertical policy diffusion for local environmental innovation into three types: “Central-Guided,” “Central-Society Driven,” and “Local Autonomy.” The following sections will analyze these three types of pathways in conjunction with typical cases, as illustrated in Table 7.

**Table 7:** Configuration analysis of vertical policy diffusion for local environmental innovation.

Antecedent Conditions	Central-guided type	Central-society driven type		Local autonomous type	
	Path 1	Path 2	Path 3	Path 4	Path 5
Central Agenda Setting	∘	∘	∘	—-	∘
Recognition by Relevant Central Government Departments	⊗	—-	⨂	⨂	—-
Focal Events	—-	∘	∘	⨂	⨂
Cost and Difficulty of Policy Innovation	⨂	⨂	—-	•	•
Diffusion of Policy Innovation Among Local Governments	⨂	⨂	⨂	∘	∘
Consistency	0.829049	0.865476	0.652609	0.899687	0.916166
Raw Coverage	0.229268	0.246079	0.169356	0.323818	0.309489
Unique Coverage	0.019294	0.155703	0.078980	0.122757	0.108428
Solution Consistency	0.840107				
Solution Coverage	0.776599				

Notes: • indicates the presence of a “core condition”; ∘ indicates the presence of a “peripheral condition”; ⊗ indicates the absence of a “peripheral condition”; ⨂ indicates the absence of a “core condition”; —- means the presence of the condition variable is indifferent to the outcome.

#### Central-guided type (Path 1).

The “Central-Guided” pathway indicates that local innovation primarily relies on the influence of central political authority and the policy preferences it promotes to drive policy diffusion. Pathway one shows that the cost and difficulty of non-policy innovation and the diffusion of non-policy innovation among local governments are core conditions. Central agenda setting and the absence of recognition by relevant central government departments are peripheral conditions, while focal events do not play a significant role in the process of local policy elevation. A typical case is the “Environmental Vertical Management” policy (Case No. 3).

China’s environmental management system adopts a territorial management model of “combining vertical and horizontal management, with a focus on the latter.” Local officials often prioritize economic growth and attracting major taxpayers, which leads to lax environmental regulations on polluting enterprises. In 2003, Jiangsu Province pioneered the “Environmental Vertical Management” policy to grant substantial authority to the vertical management within environmental departments and enhance environmental law enforcement at the grassroots level. In 2015, during the Fifth Plenary Session of the 18th Central Committee of the Communist Party of China, President Xi Jinping summarized the prominent problems in the current environmental protection system, clearly signaling the country’s significant attention to these problems. In 2016, the General Office of the CPC Central Committee and the General Office of the State Council jointly issued the “Guiding Opinions on Pilot Work for the Vertical Management System Reform of Environmental Monitoring, Supervision, and Law Enforcement Below the Provincial Level,” marking the official launch of the nationwide vertical management reform below the provincial level. The entire upward diffusion process took only 14 years, demonstrating that even with fewer satisfied conditional variables, the time required for bottom-up policy diffusion can be shortened if it occurs during a critical period when the central government is keen to address such problems. This illustrates that in areas focused on by the central government, the difficulty of upward policy diffusion is relatively low.

#### Central-society driven type (Path 2 and Path 3).

The “Central-Society Driven” pathway indicates that for local environmental practices to ascend to national policy, both “central conditions” and “social conditions” must exist. Approaches two and three show that central agenda setting and focal events are peripheral conditions. Under the dynamic perception of central intentions and the influence of social focal events, local governments will address local environmental issues by integrating their own experiences and governance needs, thereby being adopted by the country and completing the top-down diffusion process of the policy innovation. A typical case is “River Chief System” policy carried out in Jiangsu Province (Case No. 6).

As early as 2006, the State Council had noticed the problems of the river’s environment. The “Key Tasks for Environmental Protection during the 11th Five-Year Plan” explicitly emphasized that Taihu Lake was one of the priorities for strengthening water pollution prevention and control.In 2007, the blue algae pollution incident in Taihu Lake led to severe contamination of the entire Wuxi city’s tap water, resulting in a significant shortage of domestic and drinking water. Under the dual pressures of central agenda and social focal events, the Wuxi Municipal Government of Jiangsu Province pioneered the River Chief System to address the blue algae crisis. In December 2016, the General Office of the CPC Central Committee and the General Office of the State Council issued the “Opinions on Fully Implementing the River Chief System.” Within less than a decade, the River Chief System transitioned from a “bottom-up” autonomous institutional innovation to a “top-down” national water management strategy [36]. This demonstrates that when local environmental innovation meets both “central conditions” and “social conditions,” it accelerates its entry into the central policy agenda, further reducing the difficulty of policy diffusion.

#### Local-oriented type (Path 4 and Path 5).

The “Local-Oriented” pathway indicates that even in the absence of administrative orders from the central government, strong policy signals, or pressure from social focal events, local governments may still pursue environmental innovation to address specific local environmental problems by drawing on their own experiences and leveraging local resource endowments. A typical example is “Thousand Villages Demonstration and Ten Thousand Villages Renovation” project (Case No. 5) launched in Jiangsu Province.

The “Thousand Villages Demonstration and Ten Thousand Villages Renovation” project, abbreviated as the “Ten Million Project,” is a successful implementation of the philosophy that “Clear Water and Lush Mountains are Gold and Silver Mountains” in the rural areas of Zhejiang Province. In 2003, then-Party Secretary Xi Jinping initiated the “Ten Million Project” in Zhejiang Province, achieving significant results in improving the rural ecological environment and enhancing the life quality of rural residents. The project was planned and overseen by the provincial government, making it difficult to avoid the negative effects of fragmented authority among horizontal departments. Before the “Ten Million Project” was elevated into a national policy, only Guizhou Province had independently adopted and emulated it. In 2019, the General Office of the CPC Central Committee and the General Office of the State Council forwarded the “Report by the Central Agricultural Office, the Ministry of Agriculture and Rural Affairs, and the National Development and Reform Commission on Deeply Learning from the Experience of Zhejiang’s ‘Thousand Villages Demonstration and Ten Thousand Villages Renovation’ Project and Solidly Advancing the Improvement of the Rural Living Environment,” and issued a notice requiring all regions and departments to earnestly implement the report in accordance with their actual conditions.It is noteworthy that it took approximately 17 years for the “Ten Million Project” to transition from a local initiative to a nationally adopted policy. This indicates that while local autonomy can lead to the development of environmental innovation practices into national policies, the speed of policy diffusion is relatively slow. This configuration shows that the cost and difficulty of policy implementation are directly proportional to the time required for policy diffusion. Policies that are neither costly nor difficult are more likely to be adopted by other governments, demonstrating their advantages and eventually gaining recognition from relevant central government departments, or even attracting the attention of the central government, then making upward diffusion of the policy possible.

### Robustness test

Evaluating the robustness of the research findings requires analysis from two perspectives: First, it’s necessary to examine whether there are significant differences in consistency and coverage; second, it’s imperative to investigate whether there are subset relationships among the configurations. The robustness of the research findings can be confirmed only when there are no significant differences in consistency and coverage and subset relationships exist among the configurations [94]. In this study, after adjusting the consistency threshold value from 0.8 to 0.85, the overall consistency value changed into 0.91229 and the overall coverage value into 0.58795, indicating no significant differences. Furthermore, all configurations remained subsets of the original configurations. Therefore, the results of this study demonstrate a certain degree of robustness.

## Discussion and conclusion

### Research conclusions

This study, from the theoretical perspective of vertical policy innovation diffusion, employs fsQCA to integrate six conditional variables related to “central and societal-local” aspects of environmental innovation policy. It analyzes the multiple concurrent factors and complex causal mechanisms affecting the time differences in the bottom-up policy diffusion of local environmental innovations. Previous research mainly focused on single-case and single-factor impact mechanisms. However, the internal mechanisms of how multiple cases and multi-element synergistic interactions affect bottom-up policy innovation diffusion remain unclear. This study reveals that no single conditional element alone can determine the duration of policy diffusion and identifies the core configurations and five pathway mechanisms influencing diffusion time, thereby elucidating the process by which local environmental practices turn into national policies. The main conclusions are as follows:

First, The duration of the bottom-up diffusion of local policies is influenced by the combined effect of multiple conditional variables. The necessity test results for one single variable show that no single variable alone constitutes a necessary condition for shortening diffusion time. This indicates that the elevation of local environmental innovations to national policies is a complex issue that cannot rely solely on a single variable.

Second, There are five pathways for local environmental practices to turn into national policies, and they can be summarized into three models: Central-Guided, Central-Society Driven, and Local Autonomy. The Central-Guided model indicates that policy practices meet fewer local conditions but occur during a critical period in the area of the national concern, thereby meeting the requirements for shortening bottom-up diffusion time. The Central-Society Driven model suggests that policy practices simultaneously satisfy central and societal conditional variables, laying the foundation for the upward diffusion of local policies. The Local Autonomous model implies that the success and upward diffusion of local environmental practices mainly rely on the need of the local government to address specific issues by leveraging their own resource endowment. Due to the lack of other central and societal conditional variables, this model’s diffusion speed is generally slower.

### Research contributions

First, in the context of China, this study enhances our understanding of the resilience characteristic of its political system. Despite functioning as an authoritarian regime, China’s political system demonstrates notable resilience compared to similar nations, utilizing mechanisms such as five-year plans and policy pilots. These mechanisms not only facilitate economic development but also effectively mitigate various internal and external political and social challenges, thereby enabling a degree of adaptive governance [95]. A bottom-up model of innovation diffusion further exemplifies this resilience, enabling the central government to identify effective, low-cost problem-solving strategies. As these strategies are already tested and validated by numerous local governments, they are particularly suited for nationwide adoption, thereby enhancing the regime’s capacity to address relevant issues and reinforcing the political system’s legitimacy.

Second, this study broadens the conceptual scope of innovation diffusion theory. Innovation diffusion research, represented by scholars such as Rogers, has deepened our understanding of various elements, including the content, venues, agents, channels, and timing of innovations [46]. However, much of this research focuses on horizontal diffusion, with limited attention to vertical diffusion—particularly the dynamics of bottom-up innovation diffusion. Building on Rogers’ horizontal diffusion framework, this study adapts it to the Chinese context, proposing an analytical framework to assess vertical diffusion within Chinese local governments. Using environmental innovation diffusion as a case study, this research validates the framework and confirms its explanatory power, thus expanding our theoretical understanding of innovation diffusion to encompass not only horizontal but also vertical processes.

Third, this study elucidates the mechanisms through which local environmental protection innovations, initially informal practices within the oversight of the central government, may transition into formal national policies under specific conditions. This diffusion process involves diverse actors, institutional forms, and contexts, exhibiting various institutionalization modes. The process contributes to the understanding of institutionalization within new institutionalism, which emphasizes how informal practices can evolve into formal institutions. As described, “we can see how the practices developed by certain groups are legitimized over time, generally strengthened through their links to narratives which provide normative justifications for particular ways of acting; and work to embed practices as ‘common sense.’ Indeed, practices may eventually be elevated to the status of rules” [96].

Fourth, this study underscores the importance of central governments worldwide in expediting the diffusion of innovations, including environmental initiatives, based on robust empirical evidence. The natural diffusion rate of innovations across local governments is often slow, limiting their potential impact in the short term. Central governments can leverage their authority to systematically collect data on local innovations, accelerate their diffusion, and, at appropriate junctures, elevate them to the level of national policy. This approach enables a more rapid and effective response to critical environmental challenges.

### Research prospects

It is important to note that this study has some limitations that need to be addressed in future research. First, from a theoretical perspective, the bottom-up diffusion of local environmental policy innovations occurs within a structure-agency framework. This study primarily focuses on the structural factors shaping the vertical diffusion of local environmental innovations, neglecting the importance of agency factors [97] and critical time points [98]. Future research should include more variables such as local postings of personnel from central departments [99], city party secretaries (mayors) [100,101], environmental protection organizations [64,102], and critical time points to further test the reliability of the conclusions. Second, in terms of administrative levels and the time period, future studies should deepen the exploration of vertical diffusion of local environmental policy innovations. Due to data access limitations, this study mainly selected provincial governments as the research unit, so future research can extend the perspective down to city and county levels. Additionally, while this study focuses on “Five-Year Plans for Environmental Protection” and annual policy changes, future research could further explore by using more microscopic time units such as quarters and months. At last, this study measured the outcome variable solely based on the speed of policy diffusion, without adequately reflecting the extent of policy diffusion [48,50]. Furthermore, the study collected limited cross-sectional data, and since local environmental innovations exhibit continuous dynamics, future research can utilize big data techniques to collect more comprehensive panel data [103] and employ dynamic QCA methods for multi-period dynamic evolution analysis [104,105].

## References

[1] Folke C, Hahn T, Olsson P, Norberg J. Adaptive governance of social-ecological systems. Annu Rev Environ Resour. 2005;30(1):441–73.

[2] Olsson P, Folke C, Galaz V, Hahn T, Schultz L. Enhancing the fit through adaptive co-management: creating and maintaining bridging functions for matching scales in the Kristianstads Vattenrike Biosphere Reserve, Sweden. Ecol Soc. 2007;12(1). doi: 10.5751/es-01976-120128

[3] Termeer CJAM, Dewulf A, Van Lieshout M. Disentangling scale approaches in governance research: comparing monocentric, multilevel, and adaptive governance. Ecol Soc. 2010;15(4). doi: 10.5751/es-03798-150429

[4] Bulkeley H, Mol APJ. Participation and environmental governance: consensus, ambivalence and debate. Environ Values. 2003;12(2):143–54. doi: 10.1177/096327190301200201

[5] Fung A. Varieties of participation in complex governance. Public Adm Rev. 2006;66(s1):66–75. doi: 10.1111/j.1540-6210.2006.00667.x

[6] van Wormer K. Deliberative democracy and beyond: liberals, critics, contestations. Oxford: OUP; 2000.

[7] Hajer MA. The politics of environmental discourse: ecological modernization and the policy process. Oxford University Press; 1995.

[8] Graham J, Amos B, Plumptre TW. Governance principles for protected areas in the 21st century. Ottawa: Institute on Governance; 2003.

[9] Lockwood M. Good governance for terrestrial protected areas: a framework, principles and performance outcomes. J Environ Manage. 2010;91(3):754–66. doi: 10.1016/j.jenvman.2009.10.005 19896262

[10] Adger WN, Brown K, Tompkins EL. The political economy of cross-scale networks in resource co-management. Ecol Soc. 2005;10(2). doi: 10.5751/es-01465-100209

[11] Paavola J. Institutions and environmental governance: a reconceptualization. Ecol Econ. 2007;63(1):93–103. doi: 10.1016/j.ecolecon.2006.09.026

[12] Derkx B, Glasbergen P. Elaborating global private meta-governance: an inventory in the realm of voluntary sustainability standards. Global Environ Change. 2014;27:41–50. doi: 10.1016/j.gloenvcha.2014.04.016

[13] Mol AP. Environmental reform in the information age. Cambridge University Press; 2008.

[14] Keck ME, Sikkink KA. Activists beyond borders: advocacy networks in international politics. Cornell University Press; 2014.

[15] Lipschutz RD. Global civil society and global environmental governance: the politics of nature from place to planet. State University of New York Press; 1996.

[16] Bromley-Trujillo R, Butler JS, Poe J, Davis W. The spreading of innovation: state adoptions of energy and climate change policy. Rev Policy Res. 2016;33(5):544–65. doi: 10.1111/ropr.12189

[17] Baka J, Hesse A, Neville KJ, Weinthal E, Bakker K. Disclosing influence: hydraulic fracturing, interest groups, and state policy processes in the United States. Energy Res Soc Sci. 2020;70:101734. doi: 10.1016/j.erss.2020.101734

[18] Kalafatis SE. Socioeconomic reinvention and expanding engagement with climate change policy in American rust belt cities. Atmosphere. 2020;11(12):1327. doi: 10.3390/atmos11121327

[19] Metz F, Glaus A. Integrated water resources management and policy integration: lessons from 169 years of flood policies in Switzerland. Water. 2019;11(6):1173. doi: 10.3390/w11061173

[20] Cai W, Ye P. Local-neighborhood effects of different environmental regulations on green innovation: evidence from prefecture level cities of China. Environ Dev Sustain. 2022:1–25.

[21] Shin K. Mission-driven agency and local policy innovation: empirical analysis from Baoding, China. J Chin Polit Sci. 2017;22(4):549–80. doi: 10.1007/s11366-017-9514-7

[22] Wei D, Gu N. Haze governance, local government behavior and high-quality development of green economy: evidence from Chinese counties. Econ Sci. 2022;(4):64–77.

[23] Teets JC. The politics of innovation in China: local officials as policy entrepreneurs. Issues Stud. 2015;51(2):79.

[24] Mei C, Wang X. Political incentives and local policy innovations in China. J Chin Polit Sci. 2017;22(4):519–47. doi: 10.1007/s11366-017-9513-8

[25] Teets JC, Hasmath R, Lewis OA. The incentive to innovate? The behavior of local policymakers in China. J Chin Polit Sci. 2017;22(4):505–17. doi: 10.1007/s11366-017-9512-9

[26] Bogdanor V. The Blackwell encyclopaedia of political institutions. Wiley–Blackwell; 1987.

[27] Ran R. Understanding blame politics in China’s decentralized system of environmental governance: actors, strategies and context. China Q. 2017;231:634–61. doi: 10.1017/s0305741017000911

[28] Walker JL. The diffusion of innovations among the American states. Am Polit Sci Rev. 1969;63(3):880–99. doi: 10.2307/1954434

[29] Weible CM. Theories of the policy process, 4th edn. Routledge; 2017.

[30] Weible CM. Theories of the policy process, 5th edn. Routledge; 2023.

[31] Doerflinger R. The policy and politics of embryonic stem cell research. Natl Cathol Bioeth Q. 2001;1(2):135–43. doi: 10.5840/ncbq20011248 12854534

[32] Volden C. States as policy laboratories: emulating success in the children’s health insurance program. Am J Polit Sci. 2006;50(2):294–312. doi: 10.1111/j.1540-5907.2006.00185.x

[33] Mintrom M. Policy entrepreneurs and the diffusion of innovation. Am J Polit Sci. 1997;41(3):738–70.

[34] Karch A. National intervention and the diffusion of policy innovations. Am Polit Res. 2006;34(4):403–26. doi: 10.1177/1532673x06288202

[35] Allen MD, Pettus C, Haider-Markel DP. Making the national local: specifying the conditions for national government influence on state policymaking. State Polit Policy Q. 2004;4(3):318–44.

[36] Kamieniecki S, Ferrall MR. Intergovernmental relations and clean-air policy in Southern California. Publius. 1991;21(3):143–54. doi: 10.1093/oxfordjournals.pubjof.a037948

[37] Hadjiisky M, Pal LA, Walker C. Public policy transfer: micro-dynamics and macro-effects. Edward Elgar Publishing; 2017.

[38] Porto de Oliveira O. Brazil exporting social policies: from local innovation to a global model. J Polit Latin Am. 2019;11(3):249–71.

[39] Berry FS, Berry WD. State lottery adoptions as policy innovations: an event history analysis. Am Polit Sci Rev. 1990;84(2):395–415. doi: 10.2307/1963526

[40] Sugiyama NB. Bottom-up policy diffusion: national emulation of a conditional cash transfer program in Brazil. Publius. 2011;42(1):25–51. doi: 10.1093/publius/pjr019

[41] Shipan CR, Volden C. Bottom-up federalism: the diffusion of antismoking policies from U.S. cities to states. Am J Polit Sci. 2006;50(4):825–43. doi: 10.1111/j.1540-5907.2006.00218.x

[42] Karch A, Rosenthal A. Vertical diffusion and the shifting politics of electronic commerce. State Polit Policy Q. 2016;16(1):22–43. doi: 10.1177/1532440015593811

[43] Chen TNS. From local practice to national policy: a study on the innovation and diffusion of local environmental governance practices. J Hohai Univ. 2023;25(3):1–12.

[44] Yang X, Manzhu L, Yunhong G, Yinhu F. Study on the diffusion mechanism of China’s carbon emission trading system: an event history analysis based on the logic of macro-micro interaction. Urban Problems. 2023;(7):43–52. doi: 10.13239/j.bjsshkxy.cswt.230705

[45] Rong Y. The alienation of environmental policy implementation in China’s grassroots government from the perspective of intergovernmental relations: An empirical study based on S Town, Jiangsu Province. Reform Econ Syst. 2013;(4):61–5.

[46] Luhao W, Hailong L. How adjacent areas integrate into regional intergovernmental cooperation system: a case study of institutional diffusion of economic cooperation in the Yangtze River Delta. Public Adm Policy Rev. 2023;12(02):4–23.

[47] Wang H, Liu X. Classification of public service policy demonstration and policy diffusion. Zhejiang Acad J. 2024;(2):50–9. doi: 10.16235/j.cnki.33-1005/c.2024.02.008

[48] Junye W. Research on the ecological environment governance and coordination mechanism of the Yangtze River economic belt in the new era. Tianjin Soc Sci. 2023;(05):53–9. doi: 10.16240/j.cnki.1002-3976.2023.05.016

[49] Mao S, Li Y, Yixuan Y. How can local policy innovations lead to national action: From an analytical perspective of policy attributes—a case study of the river chief system. J Beijing Adm Inst. 2023;(04):66–76. doi: 10.16365/j.cnki.11-4054/d.2023.04.008

[50] Liuyi Y. Research on the ecological governance community in western ethnic areas. Soc Sci. 2023;(8):97–103.

[51] Zhang J, Zhang M. How is horizontal--vertical diffusion possible: research on the innovation diffusion process of the river chief system from institutionalized perspective—analysis based on the theory-building process-tracking method. J Public Manage. 2023;20(1):57-68 171-172. doi: 10.16149/j.cnki.23-1523.20221129.001

[52] Wang L, Pang R. Temporal and spatial evolution mechanism and policy diffusion path of China’s public policy: A study of the implementation and changes of river-chief system. Chin Public Adm. 2018;(5):63–9.

[53] Wang B, Mo QH, Mo Q. The diffusion models and effects of the local environmental policy innovation—a micro-econometric evidence from the diffusion of river chief policy. Chin Ind Econ. 2020;(8):99–117. doi: 10.19581/j.cnki.ciejournal.2020.08.006

[54] Li M, Ji Y. Restraining factors and countermeasures for the development of bottom-up environmental NGOs in China. J Hebei Univ Sci Technol (Soc Sci). 2007;(1):20–4.

[55] Li M, Wang X. Characteristics and bottlenecks of the development of bottom-up environmental NGOs in China. Product Res. 2006;(12):101–3. doi: 10.19374/j.cnki.14-1145/f.2006.12.042

[56] Yu M, Zhao Q. Action strategies of grassroots environmental organizations in participating in environmental governance from the perspective of political potential and resources. J Guizhou Normal Univ (Soc Sci). 2023;(6):83–93. doi: 10.16614/j.gznuj.skb.2023.06.009

[57] Wanh H. How to cross the river by feeling the stones?— analysis of China's government's gradual reform strategy based on policy experiments. J Guizhou Normal Univ (Soc Sci). 2021;(6):112–8. doi: 10.19735/j.issn.1006-0863.2021.06.15

[58] Li J. All-round contracting in Fengyang: from local policy to a typical example of refor. CPC History Stud. 2020;(3):125–33.

[59] Rogers EM. Diffusion of innovations. Simon & Schuster; 2003.

[60] Kapoor KK, Dwivedi YK, Williams MD. Rogers’ innovation adoption attributes: a systematic review and synthesis of existing research. Inf Syst Manage. 2014;31(1):74–91. doi: 10.1080/10580530.2014.854103

[61] Beausoleil AM. Revisiting Rogers: the diffusion of his innovation development process as a normative framework for innovation managers, students and scholars. J Innovat Manage. 2019;6(4):73–97. doi: 10.24840/2183-0606_006.004_0006

[62] Pashaeypoor S, Ashktorab T, Rassouli M, Alavi-Majd H. Predicting the adoption of evidence-based practice using “Rogers diffusion of innovation model”. Contemp Nurse. 2016;52(1):85–94. doi: 10.1080/10376178.2016.1188019 27229770

[63] Kostka G, Hobbs W. Local energy efficiency policy implementation in China: bridging the gap between national priorities and local interests. China Q. 2012;211:765–85. doi: 10.1017/s0305741012000860

[64] Economy E. Environmental governance in China: state control to crisis management. Daedalus. 2014;143(2):184–97. doi: 10.1162/daed_a_00282

[65] Zheng S, Kahn ME, Sun W, Luo D. Incentives for China’s urban mayors to mitigate pollution externalities: the role of the central government and public environmentalism. Reg Sci Urban Econ. 2014;47:61–71. doi: 10.1016/j.regsciurbeco.2013.09.003

[66] Lieberthal K. China’s governing system and its impact on environmental policy implementation. China Environ Ser. 1997;1(1997):3–8.

[67] He AJ. Manoeuvring within a fragmented bureaucracy: policy entrepreneurship in China’s local healthcare reform. China Q. 2018;236:1088–110. doi: 10.1017/s0305741018001261

[68] Kostka G, Nahm J. Central–local relations: recentralization and environmental governance in China. China Q. 2017;231:567–82. doi: 10.1017/s0305741017001011

[69] Eaton S, Kostka G. Central protectionism in China: the “Central SOE Problem” in environmental governance. China Q. 2017;231:685–704. doi: 10.1017/s0305741017000881

[70] Wang Y, Li M. Study on local government attention of ecological environment governance: based on the text analysis of government work report in 30 provinces and cities (2006–2015). China Popul Resource Environ. 2017;27(2):28–35.

[71] Li Y. Government’s attention allocation and issue identification in the governance of NIMBY activism. Chin Public Adm. 2016;(9):122–7.

[72] Chen S, Meng Q. Exploring the attention allocation mechanism of Chinese political elites: Based on the 2614 written directive from the Mao Zedong chorology biography. J Public Adm. 2016;9(03):148–76.

[73] Gilad S. Political pressures, organizational identity, and attention to tasks: illustrations from pre‐crisis financial regulation. Public Adm. 2015;93(3):593–608. doi: 10.1111/padm.12155

[74] Mertha AC. China’s water warriors: citizen action and policy change. Cornell University Press; 2017.

[75] Shipan CR, Volden C. Policy diffusion: seven lessons for scholars and practitioners. Public Adm Rev. 2012;72(6):788–96. doi: 10.1111/j.1540-6210.2012.02610.x

[76] List JA, Sturm DM. How elections matter: theory and evidence from environmental policy. Q J Econ. 2006;121(4):1249–81. doi: 10.1162/qjec.121.4.1249

[77] Wu J, Zhang P. Innovation characteristics and diffusion: a multi-case comparative study. Adm Tribune. 2014;21(1):1–7. doi: 10.16637/j.cnki.23-1360/d.2014.01.002

[78] Yang D. Diffusion of policy innovation in China: a basic analytical framework. Local Gov Res. 2016;(02):3–11.

[79] Mello PA. Qualitative comparative analysis: an introduction to research design and application. Georgetown University Press; 2021.

[80] Du Y, Jia L. Configuration perspective and qualitative comparative analysis (QCA): a new approach to management research. J Manage World. 2017;(06):155–67. doi: 10.19744/j.cnki.11-1235/f.2017.06.012

[81] Ragin CC. Set relations in social research: evaluating their consistency and coverage. Polit Anal. 2006;14(3):291–310. doi: 10.1093/pan/mpj019

[82] Zhang MCW, Lan H. Research on the antecedent configuration and performance of strategic change. J Manage World. 2020;36(9):168–86. doi: 10.19744/j.cnki.11-1235/f.2020.0145

[83] Ragin CC, Strand SI. Using qualitative comparative analysis to study causal order. Sociol Methods Res. 2008;36(4):431–41. doi: 10.1177/0049124107313903

[84] Zhang MDY. Qualitative Comparative Analysis (QCA) in management and organization research: position, tactics, and directions. Chin J Manage. 2019;16(9):1312–23.

[85] Yin RK. Case study research and applications. SAGE Publications; 2017.

[86] Lo K, Li H, Chen K. Climate experimentation and the limits of top-down control: local variation of climate pilots in China. J Environ Plan Manage. 2019;63(1):109–26. doi: 10.1080/09640568.2019.1619539

[87] Flick U. Doing triangulation and mixed methods. Sage Publications Ltd.; 2017.

[88] Fiss PC. Building better causal theories: a fuzzy set approach to typologies in organization research. Acad Manage J. 2011;54(2):393–420. doi: 10.5465/amj.2011.60263120

[89] Zuo C (Vera). Promoting city leaders: the structure of political incentives in China. China Q. 2015;224:955–84. doi: 10.1017/s0305741015001289

[90] Xie L, Xie Z, Chen C. Influence mechanism of new venture creation decision of migrants based on configurational perspective. Chin J Manage. 2021;18(09):1363–70.

[91] Sun M, Zhu F, Sun X. Influencing factors of horizontal leaders’ role identity in innovation projects: a fuzzy-set approach. Nankai Bus Rev. 2020;23(04):142–53.

[92] Xiang G, Lou S, Wang J. Who are more favored? A qualitative comparative analysis of the financing availability of startup enterprises. Stud Sci Sci. 2019;37(9):1642–50. doi: 10.16192/j.cnki.1003-2053.2019.09.013

[93] Schneider CQ, Wagemann C. Doing justice to logical remainders in QCA: moving beyond the standard analysis. Polit Res Q. 2013:211–20.

[94] Greckhamer T, Furnari S, Fiss PC, Aguilera RV. Studying configurations with qualitative comparative analysis: best practices in strategy and organization research. Strateg Organ. 2018;16(4):482–95.

[95] Nathan AJ. China’s changing of the guard: authoritarian resilience. In: Critical readings on the Communist Party of China, 4 Vols. Set. Brill; 2017, pp. 86–99. doi: 10.1163/9789004302488_005

[96] Lowndes V, Roberts M. Why institutions matter: the new institutionalism in political science. Bloomsbury Publishing; 2013.

[97] Liang M. State governancy by targets: bureaucratic accountability, performance gaps, and government behaviors. Beijing: Social Sciences Academic Press (China); 2019.

[98] Tsai KS. Adaptive informal institutions and endogenous institutional change in China. World Polit. 2006;59(1):116–41.

[99] Bulman DJ. Incentivized development in China: leaders, governance, and growth in China’s counties. Cambridge University Press; 2016.

[100] Zhao L, Hu Z. Does environmental governance promote the promotion of local officials?—An empirical study based on samples of prefecture-level cities in China. China J Econ. 2023;10(2):153–74. doi: 10.26599/cje.2023.9300206

[101] Ye Z, Wu W. Attracting the remote emperor’s attention: local policy entrepreneurship in China’s policy experimentation under hierarchy. J Asian Public Policy. 2022;:1–19.

[102] Mertha A. “Fragmented Authoritarianism 2.0”: political pluralization in the Chinese policy process. China Q. 2009;200:995–1012. doi: 10.1017/s0305741009990592

[103] Zingteng F. Issue bundling: a study on the attention allocation mechanism in the construction of digital government. Tsinghua University Press; 2023.

[104] Meng Ke WB. Rethinking the “time blindness” of the qualitative comparative analysis: bringing back “time” for public management research. Chin Public Adm. 2023;(1):96–104. doi: 10.19735/j.issn.1006-0863.2023.01.11

[105] Xie Wendong WF. A configurational exploration of how institutional pressures affect local governments’ environmental governance performance—a longitudinal QCA on prefecture-level cities. Chin Public Adm. 2024;(1):28–42. doi: 10.19735/j.issn.1006-0863.2024.01.03

